# Isolation and characterization of human cells resistant to retrovirus infection

**DOI:** 10.1186/1742-4690-4-45

**Published:** 2007-07-03

**Authors:** Patrycja Lech, Nikunj V Somia

**Affiliations:** 1 Molecular, Cellular, Developmental Biology and Genetics Graduate Program, University of Minnesota, Minneapolis, Minnesota, USA; 2 Dept. of Genetics, Cell Biology and Development and the Institute of Human Genetics, University of Minnesota, Minneapolis, Minnesota, USA

## Abstract

**Background:**

Identification of host cell proteins required for HIV-1 infection will add to our knowledge of the life cycle of HIV-1 and in the development of therapeutics to combat viral infection. We and other investigators have mutagenized rodent cells and isolated mutant cell lines resistant to retrovirus infection. Since there are differences in the efficiency of single round infection with VSVG pseudotyped HIV-1 on cells of different species, we conducted a genetic screen to isolate human cells resistant to HIV-1 infection. We chemically mutagenized human HeLa cells and validated our ability to isolate mutants at test diploid loci. We then executed a screen to isolate HeLa cell mutants resistant to infection by an HIV-1 vector coding for a toxic gene product.

**Results:**

We isolated two mutant cell lines that exhibit up to 10-fold resistance to infection by HIV-1 vectors. We have verified that the cells are resistant to infection and not defective in gene expression. We have confirmed that the resistance phenotype is not due to an entry defect. Fusion experiments between mutant and wild-type cells have established that the mutations conferring resistance in the two clones are recessive. We have also determined the nature of the block in the two mutants. One clone exhibits a block at or before reverse transcription of viral RNA and the second clone has a retarded kinetic of viral DNA synthesis and a block at nuclear import of the preintegration complex.

**Conclusion:**

Human cell mutants can be isolated that are resistant to infection by HIV-1. The mutants are genetically recessive and identify two points where host cell factors can be targeted to block HIV-1 infection.

## Background

Intensive studies of the structure and function of HIV-1 encoded genes has led to the development of a number of small molecule drugs to combat HIV-1. However, the mutation rate of HIV-1 is high (about one mutation in every 3 new genomes produced [1]) which leads to the evolution of viruses that are resistant to the drug blockade. Indeed some antiviral drugs may accelerate the mutation rate of HIV-1 [1]. This necessitates the development of new drugs and strategies to combat HIV-1 infection. In this regard, a novel approach is to target cellular factors required by HIV-1 to complete its lifecycle [2]. One method of identifying cellular factors essential for retroviral infection is through genetic screening of mutagenized cells and identifying clones resistant to infection. Complementation cloning could then be used to identify genes that confer infection susceptibility to the mutant clone. The development of high titer retroviral vectors (based on MLV and HIV-1) that recapitulate the early lifecycle of retrovirus infection greatly facilitates such screens [3]. For example, Gao and Goff (1999) isolated and characterized two mutagenized rat fibroblasts clones (R3-2 and R4-7) that are resistant to infection by MLV and HIV-1 viruses [4]. The resistance phenotype in R3-2 is due to the over expression of the FEZ1 gene [5]. Consistent with the reported block in R3-2 (after reverse transcription but before nuclear entry) FEZ1 over expression presumably interferes with transport of the reverse transcription complex or pre-integration complex in the cell. Indeed this has been demonstrated for FEZ1 overexpression and intracellular trafficking of the human polyoma JC virus [6]. The mutations responsible for the resistance in the R4-7 cell line have not been identified but can be rescued by two non-protein coding RNA suppressors: an anti-sense transcript of the transcription coactivator CAPER and a central portion of the VL30 endogenous retrovirus like element [7]. The mechanisms by which these suppressors act are not known. In another study Bruce and colleagues (2005) isolated five clones from mutagenized Chinese hamster ovary (CHO) cells that are specifically resistant to murine MLV and are not resistant to HIV-1 based vectors [8]. In our laboratory we have mutagenized hamster lung fibroblast cells (V79-4) and isolated two mutants that are (i) resistant to MLV and HIV-1 infection (ii) are blocked at pre and post reverse transcription steps and (iii) are dominant and recessive for the resistance genotype [9,10]. Studies with VSVG pseudotyped retroviral vectors (that enables infection of a wide variety of cells) have revealed differences in the efficiency of single round infection in cells of differing types and species [11,12]. Therefore, to build upon and extend the rodent cell studies, and to identify cellular factors in human cells required for the early phase of infection we have executed a genetic screen in HeLa cells to isolate mutants resistant to HIV-1 infection. HeLa cells were subjected to mutagenesis and clones resistant to infection were isolated by infecting mutagenized cells with an HIV-1 vector encoding a toxic barnase gene [9]. Successful infection results in cell death enabling the isolation of rare virus resistant clones. We isolated two resistant clones designated 30-2 and 42-7. These clones are genetically recessive for the resistance phenotype. Infection of clone 30-2 is blocked at or before virus reverse transcription. Infection in 42-7 is perturbed during reverse transcription and is impaired for nuclear import of proviral DNA.

## Results

### HeLa cell mutagenesis and validation

We exposed HeLa cells to the acridine half-mustard mutagen ICR-191 which results in frameshift mutations and chromosomal re-arrangements [13]. We used a concentration of ICR-191 that killed 90% of cells and surviving cells were allowed to recover before being subjected to another round of mutagenesis. After each round of mutagenesis, the mutation efficiency was determined at the hypoxanthine guanine phosphoribosyl transferase (HPRT) locus and at the adenine phosphoribosyltransferase locus by plating in medium containing 6-thioguanine (6-TG) or diaminopurine (DAP), respectively. These drugs select against the expression of the HPRT and APRT gene products since expression of these proteins results in the incorporation of the toxic purine analogues into DNA. The genes coding for these enzymes (HPRT X-chromosome and APRT human chromosome 16) are diploid and possibly polyploid in HeLa cells [14]. Table 1 shows the kinetics of the appearance of 6-TG and DAP resistant colonies after 7 rounds of mutagenesis. These results demonstrate that the mutagenesis procedure affected all alleles of diploid test loci HPRT and APRT in a significant portion of the cell population (1 in 10^6^) and validated the efficacy of our mutagenesis protocol.

**Table 1:** Rounds of mutagenesis to generate mutations at diploid loci.

	6-thioguanine resistant (HPRT-) colonies per 10^7 ^cells	Diaminopurine resistant (APRT-) colonies per 10^7 ^cells
Spontaneous	0	0
Round 1 mutagenesis	NA	0
Round 2 mutagenesis	NA	0
Round 3 mutagenesis	NA	0
Round 4 mutagenesis	NA	0
Round 5 mutagenesis	NA	1
Round 6 mutagenesis	31	10
Round 7 mutagenesis	26	31

NA = not assayed

Appearance of Diaminopurine and 6-thioguanine resistant colonies examined at each round of mutagenesis with ICR-191. Mutagenized HeLa cells were selected in the presence of 6-thioguanine (6-TG) and Diaminopurine (DAP) to isolate APRT (-) and HPRT (-) colonies respectively, which serve as indicators of mutagenesis at diploid loci.

### Isolation of cell clones resistant to infection by HIV-1

**Figure 1:**
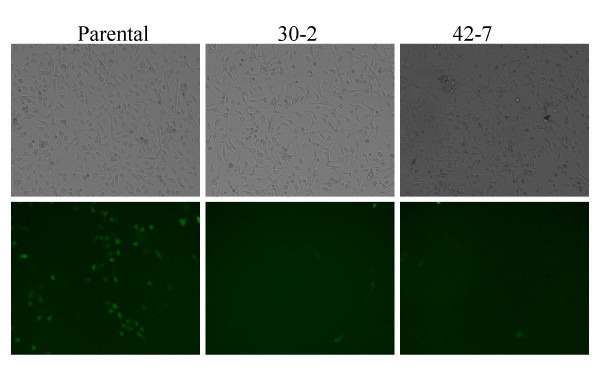
**Infection of parental and mutant HeLa cells (30-2 and 42-7) cells with an HIV-EGFP vector at a moi = 0.5**. Transmission light phase microscopy of cells is illustrated in the top panel and the corresponding field with fluorescence microscopy is illustrated in the bottom panel.

The mutagenized round 6 HeLa cells were multiply infected with a VSVG pseudotyped HIV-1 Barnase vector [9] to select for mutants that were resistant to infection. Barnase expression results in apoptotic cell death, therefore cells that survive after incubation with virus have simply escaped infection, are mutant in expression of the barnase gene or are resistant to infection by the HIV-1 vector. A total of 10^7 ^round 6 mutagenized Hela cells were infected with an HIV-1 barnase vector at a moi ≤ 2, eight times on consecutive days. Cell death became apparent on day 3 and since we infected with the same volume of virus on subsequent days the effective moi increased on subsequent infections. Cells that survived the selection were isolated and expanded. We expanded 119 clones and infected with a VSVG psuedotyped HIV-1 viral vector transducing EGFP (HIV-1 GFP/VSVG). Infection efficiency was initially semi-quantified visually by examining cells under an inverted fluorescence microscope and comparing cell clones to wild-type cells and to each other. Two clones (30 and 42) were chosen for further analysis on the basis of their resistance to infection and growth rates similar to the mutagenized round 6 HeLa cells (parental population). Each clone was further subcloned to ensure that the line is truly clonal and stable for the resistance phenotype. Subclones that displayed the latter qualities were designated 30-2 and 42-7. The variation between subclones was 2-fold with respect to infection by HIV GFP. The relative efficiency of infection of the clones is visually illustrated in Figure 1.

### Growth rates of parental and mutant cells and extent of HIV integration

**Figure 2:**
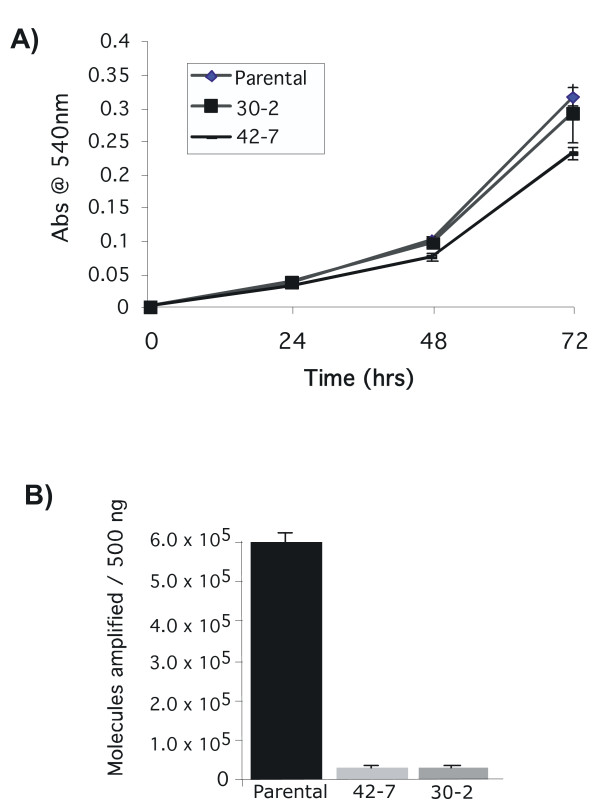
**Growth rates of parental and mutant cells and extent of HIV integration. (A) Growth rates. **Parental and mutant 30-2 and 42-7 cells were seeded and growth measured over time with the MTT assay. **(B) HIV-1 integration**. The extent of integrated HIV-1 vector was measured by infection of cells at moi = 1. The cells were passaged 3 times and the quantity of stable HIV-1 DNA was measured by quantitative real time PCR.

We tested if the refraction to infection could be explained by differences in the growth rates between parental and mutant 30-2 and 42-7 cells. Figure 2A illustrates that the growth rates are not significantly different between the parental and mutant cells. To examine if the defect in infection was in the early stages of the life-cycle we next examined the extent of integration of HIV-1 DNA after infection of parental and mutant cells. Figure 2B illustrates the results of a qPCR analysis for HIV-1 in genomic DNA of parental and mutant cells that were infected (at an moi = 1) and passaged 3 times before DNA extraction. This analysis reveals over a 10-fold reduction in the amount of DNA integrated into the genome of mutant cells.

### Clones 30-2 and 42-7 are resistant to MLV and HIV-1 infection

**Figure 3:**
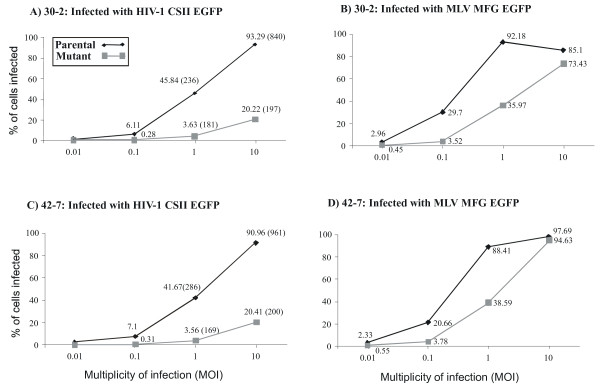
**Clones 30-2 and 42-7 are resistant to MLV and HIV-1 infection**. Parental (diamond point and black line), 30-2 and 42-7 (square point and grey line) cells were infected with HIV GFP/VSVG (2A, C) and MLV GFP/VSVG (2B, D) at an increasing moi of 0.01, 0.1, and 1. Data is expressed as % of GFP positive cells determined by fluorescence cytometry.

We then quantified the resistance to infection of clones 30-2 and 42-7 by fluorescence cytometry relative to the parental population. We further determined if the resistance is specific to HIV-1 or common to other evolutionarily distinct retroviruses such as murine leukemia virus (MLV). The clones and parental round 6 cell lines were infected with VSVG pseudotyped HIV-1 EGFP or MLV EGFP vector at an moi of 0.01, 0.1, 1 and 10 (the moi were determined by infection of non-mutagenized HeLa cells). This range of moi ensured that the infection was in a linear range for quantification. Infections were analyzed by fluorescence cytometry 72 hours later. Typical results from this analysis are illustrated in Figure 3. The range is considered linear where increase in moi yields a corresponding increase in the number of cells infected and the geometric means of fluorescence (a measure of multiple infections) are also comparable. By this analysis clone 30-2 is 12 fold less infectable with the HIV-1 vector (Fig 3A, at an moi = 1) and approximately 10 fold less infectable with the MLV vector (Fig 3B at an moi = 0.1). Clone 42-7 is 10 fold less infectable by HIV-1 EGFP (Fig 3C at moi = 1) and 5 fold less infectable by MLV EGFP (Fig 3D at an moi = 0.1). This phenotypic analysis of HIV GFP infection correlates with the molecular analysis of the extent of integration with a 10-fold reduction in the mutant cells (Fig. 2B). Strikingly both clones remain resistant to HIV-1 at high MOI whereas they become almost as sensitive to MLV as wild type cells. This might indicate a greater dependence for HIV-1 on, or sensitivity to, the factors that have been altered by the mutagenesis.

### Resistance is independent of the reporter and is not a defect in gene expression of the reporter

**Figure 4:**
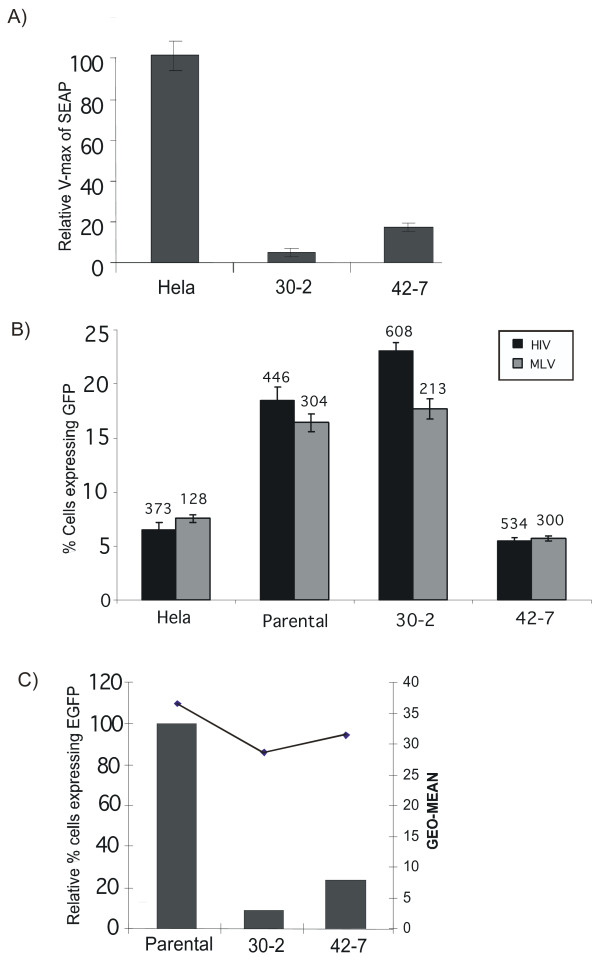
**HeLa mutants are resistant to infection. (A) Resistance to infection is independent of the reporter. **Clones 30-2 and 42-7 were infected (moi ~ 0.5) with HIV-1 viral vector transducing the gene for secreated alkaline phosphatase (SEAP). The amount of SEAP released by infected cells was measured 72 hours latter and is expressed as the V_max _of SEAP activity. **(B) Resistance to infection is not due to a defect in reporter gene expression. **HeLa, Parental (Round 6 mutagenesis), 30-2 and 42-7 cell lines were transfected with an HIV vector plasmid where EGFP expression is dictated by the human EF1α promoter and with the MLV viral vector where EGFP expression is controlled by the CMV promoter. Although the cells differ in their transfection efficiencies they show comparable levels of GFP expression. The x-geo mean intensity of EGFP expression is indicated above each bar. **(C) Resistance to infection is independent of receptor use and HIV-1 accessory proteins**. Parental, 30-2 and 42-7 were infected with a fully accessorized HIV-1 viral vector psuedotyped with a 10A1 envelope, which uses a pH independent pathway of entry. The line graph depicts that x-geo mean intensity of each sample.

We next examined if the resistance phenotype is due to a defect of the reporter or due to defects in expression of the reporter. To verify that the resistance is not due to a defect of the EGFP reporter used we next infected the parental cells and the 30-2 and 42-7 mutant clones with an HIV-1 based vector transducing a gene coding for secreted alkaline phosphatase (SEAP). Infection was quantified by the amount of SEAP secreted into the media by infected cells [15]. Subclone 30-2 was 20 fold resistant and 42-7 was 6 fold resistant to HIV-1 viral vector infection using this assay (Figure 4A). We conclude that the observed resistance to infection is independent of the reporter used. We next investigated if the observed resistance is due to defects in expression of the reporter. We transfected wild-type and mutant cells with the HIV-1 EGFP vector (in which the human EF1α promoter dictates EGFP expression) or the MLV vector (where EGFP expression is directed by the early human CMV promoter). Fig. 4B illustrates the results from this experiment. While the transfection efficiency can vary between cell types (compare HeLa cells to 30-2 cells) the overall gene expression (as determined by mean fluorescence intensity) is similar between HeLa and mutant cells for both the HIV-1 and MLV EGFP vectors. Hence the block seen on infection is not due to alterations in gene expression in the 30-2 and 42-7 mutant cells.

### Resistance is independent of receptor use and accessory factors

Lentivirus encoded accessory factors can mitigate infection of certain cell types [16,17]. The HIV-1 packaging construct used in this study, ΔNRF retains Tat, Rev and Vpu coding [18]. To test the effect of Nef, Vif and Vpr (accessory proteins that are packaged into virons [19-21]) we generated vectors with packaging plasmids providing all these accessory proteins. Furthermore to determine if the mutant clones are deficient for VSVG mediated entry, we pseudotyped the HIV-1 based vectors with the MLV amphotropic envelope, 10A1. The envelope protein 10A1 of the amphotropic retrovirus binds to phosphate transporter proteins Pit-1 or Pit-2 [22] and enters using a pH independent pathway [23], while VSVG is thought to bind a phospholipid [24] and infects using a pH dependent pathway [23]. Figure 4C illustrates the analysis from infection of wild type and mutant cells using 10A1 pseudotyped HIV-1 virus produced in the presence of all HIV-1 accessory proteins. Both mutant cell lines retain the resistance to infection. We conclude that (i) HIV-1 accessory proteins cannot rescue the resistance to infection in the 30-2 and 42-7 mutant cell type and (ii) the resistance is independent of the receptor used for entry or the route of entry (pH dependent or independent pathways).

### Analysis of proviral DNA synthesis in mutant cells

**Figure 5:**
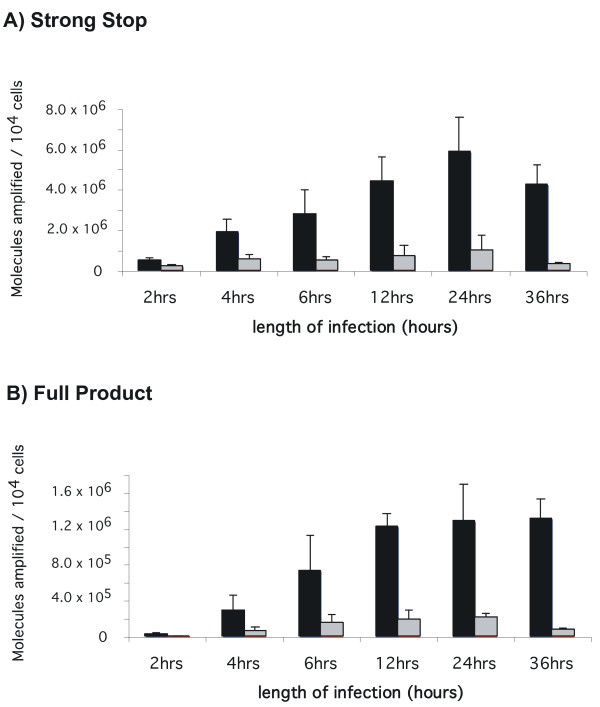
**Kinetics of proviral DNA synthesis in wild-type and 30-2 cells**. Parental (black bars) and 30-2 (grey bars) cells were infected with HIV-1 GFP/VSVG at a moi of 0.5 and viral DNA products quantified at the indicated times. (A) amplification of strong stop early product and (B) amplification of late full product. The absence of contamination was confirmed by the failure to amplify viral replication intermediates from water and a heat inactivated viral vector (data not shown).

**Figure 6:**
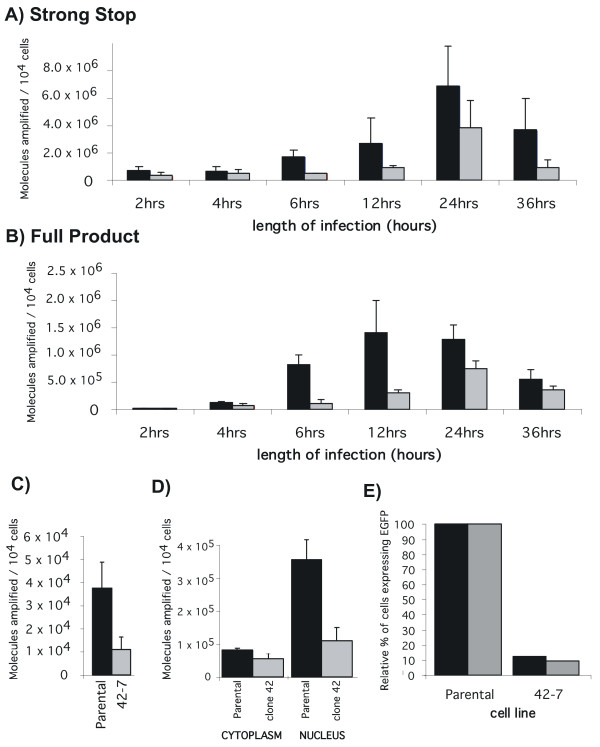
**Synthesis and localization of proviral DNA in wild type and mutant 42-7 cells**. Parental (black bars) and 42-7 (grey bars) cells were infected with HIV-1 GFP/VSVG at a moi of 0.5 and viral DNA products quantified at the indicated times. (A) amplification of strong stop early product; (B) full product; (C) 2LTR circles 36 hours after infection; (D) biochemical fractionation of cytoplasmic and nuclear extracts and measurement of the amount of full product 36 hours after infection in parental (black bars) and 42-7 (grey bars) cells; (E) Parental and 42-7 cells were infected with HIV/GFP/VSVG (black bars) or with a viral vector generated with a mutant integrase (grey bars) to examine expression 72 hrs latter of the EGFP reporter from circles and other unintegrated viral products.

To further characterize the block to infection we next followed the formation of viral DNA products over time in infected wild-type and mutant clones. Subclone 30-2, 42-7 and the parental cell line were infected and total DNA extracted at different times post infection. Viral DNA was amplified using real-time qPCR and primers were used to amplify specific reverse transcription intermediates by hybridizing to particular regions of the viral genome. This allows discrimination of strong stop and full products of the reverse transcription process. The number of molecules of reverse transcription product formed was calculated from the quantity of PCR product by reference to a standard curve. The results of this analysis are illustrated in Figure 5 for 30-2 and Figure 6 for 42-7. qPCR analysis of subclone 30-2 revealed that over a 36 hour period the strong stop primers amplified 2 to 16 fold less initial minus strand DNA product when compared to the control cells (Figure 5A). A similar trend was revealed by the full product primer sets (figure 5B), suggesting that the virus is blocked before or at the stage of reverse transcription. In clone 42-7, the formation of viral DNA intermediates is also initially decreased – on average a 2-fold decrease in the amount of products formed for the strong stop (Figure 6A). This decrease is also apparent at earlier time points for the full-product. However the difference is less apparent at the latter (36 hr) timepoint (Figure 6B). We conclude from this that the synthesis of proviral DNA is retarded in 42-7 cells. Notably, even though the molecular analysis reveals that there is near equivalence of proviral DNA synthesis this does not correlate to the titer of virus on 42-7 cells (10 fold less than wild type cells, see Figure 3) or the level of integration (Figure 2B). Indeed the titer does not increase even if infection (% EGFP infected cells) is measured at 144 hrs rather than 72 hrs (data not shown). We conclude from this that one of the blocks to infection in 42-7 cells is due to a slower completion or aberrant reverse transcription.

### 42-7 cells are further impaired for nuclear entry of viral DNA

The accumulation of 2 LTR circles is a product of intra-molecular ligation of the linear reverse transcription product and can be a surrogate molecular marker for nuclear entry of viral DNA [25]. Hence we next asked if the nuclear accumulation of viral DNA was impaired in 42-7 cells. PCR primers were used to probe the extent of 2LTR circle accumulation. Results of this analysis (Fig 6C) reveal that accumulation of 2LTR circles is impaired in 42-7 cells suggesting a defect in nuclear entry of viral DNA. However the ratio of linear and 2 LTR circles has been reported to be altered by certain cell factors (i.e RAD 52 [26]). Hence to exclude this possibility we quantified HIV DNA 36 hours after infection in cytoplasmic and nuclear extracts of parental and 42-7 cells. This analysis (Fig 6D) reveals up to a 4 fold difference in the accumulation of HIV DNA in nuclear extracts between parental and mutant cells. This difference correlates with the deficiency in the accumulation of 2 LTR circles in 42-7 cells (Fig 6C). To examine expression of the EGFP reporter from these circles and other unintegrated viral products we infected cells with a viral vector generated with a mutant integrase deficient helper plasmid. This analysis (Fig 6D) reveals EGFP is expressed in only 10% of cells compared to wild-type parental cells which is comparable to integrase proficient vector. We conclude from this analysis that although reverse transcription may reach completion (albeit slower, see Fig 6B) that the product of this reaction is not comparably detected in the nucleus.

### Resistance to infection in 30-2 and 42-7 cells is recessive

**Figure 7:**
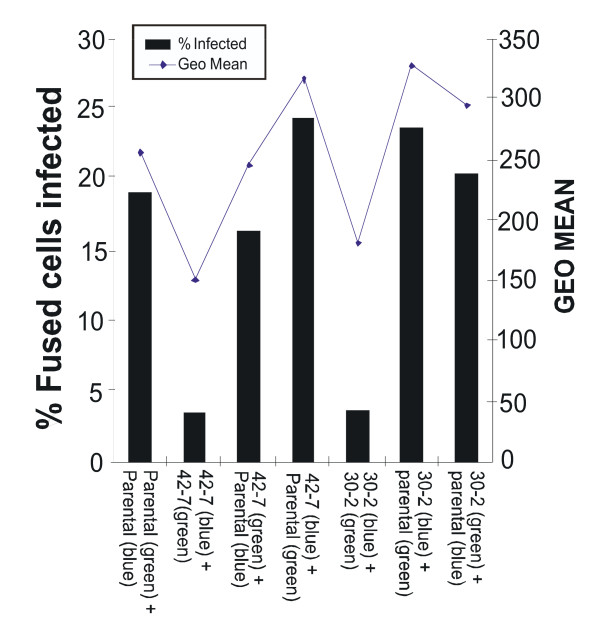
**42-7 and 30-2 are recessive mutants**. Parental, 30-2 or 42-7 cells were labeled with the membrane dyes Oregon Green or Vybrant DID that mark cells green and blue respectively. The cells were then mixed (either as self-self or as parental and mutant combinations) and fused with polyethelene glycol (PEG). The fused cells were infected with HIV-1 virus transducing DsRed and analyzed by flourecence cyometry. The bar graph illustrates the percentage of fused cells expressing DsRed and line graphs the geometic mean intensity of DsRed expression.

We next asked if the mutations conferring resistance to infection in the mutant cells were dominant or recessive. To address this question we performed cell fusion experiments between wild type parental and mutant cells. Figure 7 illustrates an example of such an analysis. Parental, 30-2 or 42-7 cells were labeled with the membrane dyes Oregon Green or Vybrant DID that mark cells green and blue respectively. These differentially marked cells were then mixed (either as self-self or as parental and mutant combinations) and fused by addition of polyethelene glycol (PEG). The fused cells were then infected with a dsRED marked HIV-1 virus and the homo and hetrokaryons analyzed by fluorescence activated cytometry. While mutant homokaryons exhibit a resistance to infection compared to parental homokaryons (3.5% for 42-7 to 42-7 fusions; 3.7% for 30-2 to 30-2; and 20% for parental fusions) this resistance is rescued in the parental and mutant hetrokaryons (16.4% and 24.5%) in the reciprocal staining experiments for 42-7 and parental fusions and 23.8% and 20.5% for the 30-2 and parental fusions). In repeat experiments we routinely observed that in the reciprocal dyeing experiments the rescue is more pronounced when the mutant cells are labeled with Vybrant DID. We conclude from this experiment that the mutations causing the resistance in 30-2 and 42-7 are recessive.

## Discussion

In this study we report on the isolation of two human clones that are resistant to infection by HIV-1 and MLV viruses. Somatic cell mutagenesis and complementation cloning is a powerful approach for the identification of host cell factors involved in the retroviral lifecycle. An early application of this approach (by Hillman and colleagues (1990) [27]) used EMS mutagenesis in a screen to derive human T-cell clones with varying CD4 expression levels. This group also characterized some mutants with normal CD4 expression but a reduced capacity for HIV-1 replication due to defective in NFκB signaling [28,29]. These mutants were from a screen that initially targeted CD4 expression. Gao and Goff (1999) [4] isolated mutant cell clones on the basis of resistance to MLV and observed that the cells were also resistant to HIV-1 vectors. In contrast Bruce et al., (2005) [8] isolated clones from mutagenized Chinese Hamster Ovary cells that are uniquely resistant to MLV infection but are infected normally by HIV-1 and ASLV vectors. We have isolated clones from mutagenized hamster lung fibroblasts (V79-4) cells that are refractory to both MLV and HIV-1 vectors ([9,10]). Taken together these studies imply that evolutionary distant retroviruses utilize common and distinct host cell factors. In this study we have extended these observations to human cells. Here we report two clones that are resistant to both HIV and MLV vectors although the resistance is more pronounced for HIV-1 (Figure 3). We note the clones that we and others have isolated are not entirely resistant to infection but rather refractory to infection. There are several possible hypotheses that may explain this: (i) Gene mutations that would make cells totally resistant are also lethal for cell viability (ii) the screens are not saturating and totally resistant clones have been missed (iii) HIV-1 and MLV use redundant, but saturable pathways for infection, and these clones are mutant in only one pathway and (iv) the clones are "leaky" and produce reduced amounts of protein needed for infection. We demonstrate that the resistance to infection is not at the level of gene expression by transfection of the vector DNA into mutant cells (Figure 4B). Notably, isolated clones vary in transfection efficiency compared to the parental population and hence interpretation of these results are in the context of both transfection efficiency and level of expression (as judged by the geometric mean fluorescence). All the studies reported thus far have utilized the VSVG envelope to pseudotype MLV and HIV-1 vectors during the selection procedure. To date no clones have been reported that are due to an entry block to VSVG. The resistance in the two human mutants reported here is also independent of the envelope used (Figure 4C). We further characterized the blocks to infection and identified a block at or before reverse transcription in the 30-2 clone (Figure 5). We also identified a block in 42-7 cells with retarded kinetics of reverse transcription with a subsequent block to nuclear import (Figure 6). There is a disparity in the number of molecules in the nucleus (approximately 1/4 of wild-type in the mutant cells), the expression and integration analysis (Fig 6D and Fig 2B) reveals a log difference in infection between wild type and 42-7 cells. While this may be due to differences in the levels of detection between the PCR analysis and fluorescence cytometry, the slower synthesis and reduced nuclear import suggests that the products of the reverse transcription reaction may be aberrant. We are currently examining this hypothesis. Although we and others have identified blocks pre and post reverse transcription the 42-7 mutant represents a novel phenotype in the slower kinetic of reverse transcription. Cell fusion experiments have also allowed us to conclude that the block to infection is recessive and can be rescued by fusion with wild-type cells (Figure 7). This analysis does not suggest a mechanism for the resistance to infection. For example it is possible that a mutation of a transcriptional repressor may activate the expression of a restriction factor [5]. However this experiment does suggest that complementation cloning by transfer of cDNA libraries derived from wild-type cells [30,7] is a feasible approach and should yield novel host cell factors involved in the early stages of retrovirus infection.

## Conclusion

Human cell mutants can be isolated that are resistant to infection by HIV-1 and MLV. The mutants are genetically recessive and blocked at or before reverse transcription and in nuclear import.

## Methods

### Tissue culture

293T cells, HeLa and derived cell lines (30-2 and 42-7) were maintained in Dulbecco's modified Eagle's medium, DMEM (Cellgro) supplemented with 10% Fetal Bovine serum, FBS (Gemini Bioproducts). During heterokaryon experiments HeLa and derived cells lines were maintained in DMEM without phenol red supplemented with 20% FBS.

### Virus production

MLV and HIV-1 vectors were generated by transient transfection of multiple plasmids into 293T cells as described previously [30,31]. Briefly, for MLV based vector 10 μg of CMVgp, 5 μg of pMDG and 15 μg of vector DNA were transfected using the method of Chen and Okayama [32]. 72 hrs after transfection virus was collected, filtered through a 0.45 μ membrane and stored at -80°C. HIV-1 based vectors were similarly generated using 10 μg of ΔNRF (a kind gift from Dr. Tal Kafri, [18]), 5 μg pMDG [31] or pRK510A1 (N.S unpublished) and 15 μg vector DNA (CSII EGFP, CSII DsRed, CSII Barnase [9] or CSII SEAP (N.S. unpublished)). An integrase-defective packaging plasmid ΔR8.2 (INT-) with a point mutation in the integrase (D64V) was kindly provided by Dr. Tal Kafri. Viral titers for EGFP transducing vectors were determined by infecting 10^5 ^HeLa cells with serial (10 fold) dilutions of the vector preparation. The medium was changed after 12 hours incubation of the viral vector with the cells, and the extent of EGFP expression was quantified 72 hours after infection by flow cytometry on a Becton-Dickinson FACScalibur. HIV-1 based viral vectors utilized for qPCR analysis were treated with 25 U/ml DNaseI at room temperature for 1 hour.

### Mutagenesis of HeLa cells

10^8 ^HeLa cells were mutagenized for 10 hours with 10 μg/ml ICR-191 (Sigma), followed by a media change and a recovery period. Mutagenesis was repeated for 7 rounds. After each round an aliquot (10^7 ^cells) was incubated with 6-thioguanine (10 mg/ml) or 2-aminopurine (50 mg/ml) and resistant clones were quantified when visible colonies appeared. Aliquots of cells were frozen at -80°C after each round of mutagenesis.

### Screening of HIV-1 resistant clones

HeLa cells that were mutagenized for 6 rounds were infected 8 times with a VSVG pseudotyped HIV-1 vector encoding Barnase [9] at an initial moi = 2, on 8 consecutive days. The 119 colonies that survived the selection were isolated and resistance to infection was assessed by infecting with VSVG pseudotyped HIV-1 and MLV viral vectors transducing EGFP. The efficiency of infection was assessed visually using an inverted fluorescence microscope, and the most resistant clones (as compared to the wild-type parental cells and to each other) were selected for further study. The clones were further sub-cloned by limiting dilution to ensure that the clones were homogeneous, and that the resistant phenotype was stable.

### Growth analysis

1 × 10^4 ^cells were seeded in 24 well plates and at given time points viable cells were measured using the MTT assay [33]. Briefly, at given time points media was replaced with 500 μl 1X MTT solution and cells were incubated for 1 hr at 37°C and the MTT solution was removed. Cells were lysed in acetic isopropanol (400 ul Isopropanol + 40 mM HCl) and the absorbance measured at 540 nm.

### Flow Cytometry analysis

Infected or transfected cells expressing EGFP or Ds Red proteins were quantified by Fluorescence cytometry on a Becton-Dickinson FACScan and analyzed using Becton-Dickinson CellQuest 3.1 software at the Flow Cytometry Core Facility of the University of Minnesota Cancer Center.

### Secreted alkaline phosphatase (SEAP) assay of viral infection

10^5 ^cells were seeded in triplicate in 6 well plates and infected with a VSVG psuedotyped HIV-1 vector transducing SEAP (CSII-EF-SEAP, N.S, unpublished). 12 hrs post infection the media was changed to remove the viral supernatant. 72 hrs post infection SEAP activity within the media of infected and uninfected cells was assayed as previously described [34]. Briefly, media was collected from each well and heated at 65°C to inactivate endogenous phosphatases. Serial dilutions of the heat inactivated samples were made in DMEM. Samples were mixed at a 1:1 ratio with 2 × SEAP buffer (2 M Diethanolamine; 1 mM MgCl2; 20 mM L-homoarginine). The substrate (120 mM p-nitrophenol phosphate) was dissolved in 1 × SEAP buffer and 1/10 sample volume was added to each sample. The kinetics of the reaction was measured as absorbance at 450 nm every 5 min for 30 min at 37°C using a plate reader (Bio-Tek Synergy HT).

### Cell fusion assay

Cells were stained with Oregon Green (Invitrogen probes Cat # O34550) or with Vybrant DID (Invitrogen probes Cat # V22887) for 15 min according to the manufactures protocol. The stained cells were gently washed 3 times with PBS buffer and between each wash the cells were incubated for 10 min at 37°C. The stained cells were left to recover for 4 hrs in DMEM (without phenol red) supplemented with 10% FBS. Cell fusions were performed by removing the cells from the plate with a non-trypsin dissociation media and self-self or parental and mutant cells were mixed in 15 ml conical tubes (Falcon) and pelleted by centrifugation for 5 min at 500 g. The pelleted cells were incubated in 1 ml of a sterile PBS solution containing 50% Polyethelene glycol (PEG 3000–3700 Da) (Sigma) and 2% Glucose for 45 seconds. The cell suspension was then diluted with 1 ml of PBS and incubated for another 45 seconds. The PEG solution was further diluted with 3 mls of wash buffer (PBS + 2% FBS) before being centrifuged at 500 g for 5 min. Gentle resuspension of cells in wash buffer and pelleting of cells by centrifugation was repeated 3 times before resuspending the cells in DMEM media without phenol red supplemented with 20% FBS. Cells were allowed to recover and settle for 6–8 hours in 10 cm tissue culture plates before being infected with HIV vector transducing DsRed. 48 hours post infection the cells were analyzed by fluorescence cytometry using 4 color differentiation on a Becton-Dickinson FACSCalibur. Background leakage through the channels was compensated by subtraction of the background value from all samples.

### Reverse transcription product qPCR assay

3.5 × 10^5 ^cells were plated into 6 well dishes and infected at a moi= 0.5 with DNaseI treated viral supernatant. To control for DNA contamination, DNaseI treated virus was placed in a boiling water bath for 30 minutes to serve as a heat inactivated sample control. Cells were incubated with virus for 6, 12, 24, or 36 hours. Controls consisted of uninfected cells or cells infected with heat inactivated virus for 36 hours. Infection was stopped by harvesting the cells and washing them with PBS buffer. Total cell lysate was prepared by resuspending the cell pellet in lysis buffer (Tris pH 8.0, 25 mM EDTA pH 8.0, 100 mM NaCl, 1% Triton X-100, and 2 mg/ml proteinase K) and incubating at 55°C overnight. The next day, the proteinase K was heat inactivated at 95°C for 15 minutes. Lysates were used directly for PCR analysis. The following primers were used for qPCR [35] : 5' β-actin-ATC ATG TTT GAG ACC TTC AA, 3' β-actin-AGA TGG GCA CAG TGT GGG T, LTR9 – GCC TCA ATA AAG CTT GCC TTG, 5NC2 – CCG AGT CCT GCG TCG AGA GAG C, AA55 -CTG CTA GAG ATT TTC CAC ACT GAC, LTR8 TCC CAG GCT CAG ATC TGG TCT AAC. LTR9 and AA55 were used to amplify the strong stop product, LTR9 and 5NC2 amplified the full product and LTR8 and LTR9 amplified the 2 LTR circle products. Quantitative PCR reactions using SYBR green were performed using a Biorad iCycler equipped with an optical module and BioRad SuperMix (without ROX) following the manufacturer's protocol. Cycling conditions used were 95°C for 3 min, followed by 35 cycles of 95°C 30s, 58°C 30s, and 72°C 30s, and a final extension (5 minute 72°C) to complete all the PCR products. Quantification of the amount of DNA was calculated from the cycle threshold (C_T_) determined using the Bio-Rad software. The melt curve as well as analysis of the PCR products by agarose gel electrophoresis confirmed the presence of one product at the expected size (data not shown). DNA input was controlled by qPCR amplification of a fragment of the β-actin gene. The number of molecules amplified in test samples was extrapolated from a standard curve generated from the viral vector DNA of known concentration for strong stop and full product primer sets. Standard curves for the 2LTR product was generated using PCR generated 2LTR product that was purified to homogeneity and quantified by spectrometry.

### Analysis of integrated HIV DNA

Parentals, 30-2 and 42-7 cells were infected with HIV GFP at moi = 1. Virus was removed from the cells 24 hrs latter and fresh media added. After three passages (with each passage constituting a 1/10 split) that dilute out all non integrated viral DNA, cells lysates were prepared as outlined above. 500 ng of DNA was used as a template to amplify full product by real-time quantitative PCR as outlined above.

### Nuclear and Cytoplasmic separation

Nuclear and cytoplasmic fractions were isolated as previously described [36]. Briefly, 3 × 10^5 ^parental and 42-7 cells were infected with HIV GFP vector at an MOI = 0.5 and 36 hrs latter washed with PBS and lysed on ice with 100 μl hypotonic buffer (10 mM Tris, pH 7.5; 10 mM NaCl; 1 mM EDTA; 100 g of digitonin per ml) for 5 minutes. The lysates were centrifuged for 5 min at 1,500 rpm (Eppendorf microfuge) and the pelleted nuclear fraction was resuspended in 100 μl hypotonic buffer. The supernatant was further centrifuged for 5 min at 13,000 rpm and the supernatant constituted the cytoplasmic fraction. Real-time quantitative PCR was used to detect full product in the nuclear and cytoplasmic fractions as outlined above.

## Abbreviations

HIV-1, human immunodeficiency virus type 1; MLV, murine leukemia virus; VSVG, vesicular stomatitis virus G protein; HPRT, hypoxanthine guanine phosphoribosyl transferase; APRT, adenine phosphoribosyltransferase; 6-TG, 6 thioguanine; DAP, diamino purine; EGFP, enhanced green fluorescent protein; SEAP, secreted alkaline phosphatase; EF1α, elongation factor 1 alpha; CMV, cytomegalovirus; LTR, Long terminal repeat; PEG, polyethylene glycol; EMS, ethylmethanesulfonate; ASLV, Avian sarcoma and leukosis virus.

## Competing interests

The author(s) declare that they have no competing interests.

## Authors' contributions

PL and NS conceived of and executed the experiments. PL and NS wrote the manuscript. All authors read and approved the final manuscript.
